# Cadmium Stress Reprograms ROS/RNS Homeostasis in *Phytophthora infestans* (Mont.) de Bary

**DOI:** 10.3390/ijms21218375

**Published:** 2020-11-08

**Authors:** Joanna Gajewska, Nur Afifah Azzahra, Özgün Ali Bingöl, Karolina Izbiańska-Jankowska, Tomasz Jelonek, Joanna Deckert, Jolanta Floryszak-Wieczorek, Magdalena Arasimowicz-Jelonek

**Affiliations:** 1 Department of Plant Ecophysiology, Institute of Experimental Biology, Faculty of Biology, Adam Mickiewicz University Poznań, Uniwersytetu Poznańskiego 6, 61-614 Poznań, Poland; joanna.gajewska@amu.edu.pl (J.G.); nurafifahaz92@gmail.com (N.A.A.); karolina.izbianska@amu.edu.pl (K.I.-J.); Joanna.Deckert@amu.edu.pl (J.D.); 2 Department of Biology, Eskisehir Technical University, 26555 Eskisehir, Turkey; ozgunalibingol@gmail.com; 3 Department of Forest Utilization, Poznań University of Life Sciences, Wojska Polskiego 71A, 60–625 Poznań, Poland; tomasz.jelonek@up.poznan.pl; 4 Department of Plant Physiology, Poznan University of Life Sciences, Wołynska 35, 60-637 Poznań, Poland; jolanta.floryszak@up.poznan.pl

**Keywords:** reactive oxygen and nitrogen species, fungal-like organism, pathogenicity, late blight disease, heavy metal pollution

## Abstract

Heavy metal pollution causes many soils to become a toxic environment not only for plants, but also microorganisms; however, little is known how heavy metal contaminated environment affects metabolism of phytopathogens and their capability of infecting host plants. In this study the oomycete *Phytophthora infestans* (Mont.) de Bary, the most harmful pathogen of potato, growing under moderate cadmium stress (Cd, 5 mg/L) showed nitro-oxidative imbalance associated with an enhanced antioxidant response. Cadmium notably elevated the level of nitric oxide, superoxide and peroxynitrite that stimulated nitrative modifications within the RNA and DNA pools in the phytopathogen structures. In contrast, the protein pool undergoing nitration was diminished confirming that protein tyrosine nitration is a flexible element of the oomycete adaptive strategy to heavy metal stress. Finally, to verify whether Cd is able to modify *P. infestans* pathogenicity, a disease index and molecular assessment of disease progress were analysed indicating that Cd stress enhanced aggressiveness of vr *P. infestans* towards various potato cultivars. Taken together, Cd not only affected hyphal growth rate and caused biochemical changes in *P. infestans* structures, but accelerated the pathogenicity as well. The nitro-oxidative homeostasis imbalance underlies the phytopathogen adaptive strategy and survival in the heavy metal contaminated environment.

## 1. Introduction

Environment contamination by heavy metals is a huge problem in many parts of the world. The rate of the global deposition of heavy metals in soil has dramatically increased over the past two centuries [1]. Therefore, it is currently a serious threat not only to plants and microorganisms, but also to all biotic interactions, in which at least one of the components is exposed to the metals [2,3]. Heavy metals may be divided into the elements, which in small amounts are essential for living organisms (e.g., chromium, iron, zinc) and the elements of an unknown physiological role (e.g., cadmium, lead, mercury), which are considered toxic for many organisms including animals, plants, and microorganisms [4]. The former group of heavy metals is called essential and the latter nonessential elements. Nonetheless, both of these groups can be toxic for living organisms in higher than the critical concentrations [5]. One of the nonessential metals that is relatively widely distributed in nature (air, sediment, soil, water) and released into the environment in the amount of about 30,000 tonnes per year is cadmium (Cd) [6]. As a highly mobile, redox-inactive metal, Cd is easily absorbed by living organisms, which can exert its negative effects related to morphological, structural, and molecular changes. Importantly, Cd-dependent gene expression could be regulated by changes in the activity of transcription factors, the modulation of micro RNA levels, and modifications in chromatin [7].

In general, metals considered as nonessential elements disrupt the proper functioning of the organism, resulting in a stress status. One of the earliest reactions to these heavy metals noted in various model organisms is reactive oxygen and nitrogen species (ROS/RNS) generation [8]. An uncontrolled accumulation of both ROS and RNS can provoke alteration of the cell redox balance resulting in oxidative/nitrative modifications of biomolecules that contribute to the heavy metal toxicity [8]. On the other hand, ROS/RNS are ubiquitous signaling molecules engaged not only in growth and development, but also in stress recognition, signal transduction, and the response to stress factors facilitating alleviation of heavy metal toxicity [9,10,11]. The final effect of ROS/RNS on the cellular environment is dependent on their turnover, which includes production and detoxification pathways [12,13].

While the mechanisms associated with the microorganism response to essential metals such as copper, iron, and zinc have been extensively studied and reviewed [14,15,16,17], the response to nonessential metals is definitely less recognized, especially with regard to fungal and fungal-like pathogens. In general, heavy metals primarily affect fungal growth and morphology [18]. These effects as a consequence to heavy metal exposure were observed e.g., in *Phanerochaete chrysosporium* Burds. [18], *Fusarium oxysporum* Schlecht. [19], *Botrytis cinerea* Pers., *Alternaria alternata* (Fr.) Keissl. [20], *Aspergillus niger* van Tieghem, and *Penicillium citrinum* Thom [21]. Moreover, in the white-rot fungus *P. chrysosporium* [13] and in filamentous yeast *Trichosporon cutaneum* Kreger-van Rij [22] heavy metals induced oxidative stress and antioxidant enzyme activity. A similar heavy metal-dependent effects were observed in fungal-like oomycetes. Briefly, the metal reduced hyphae area and radial extension in *Pythium debaryanum* Hesse, *Achlya bisexualis* Coker and Couch, *Saprolegnia delica* Coker, *Dictyuchus carpophorus* Zopf., and *Phytophthora capsici* Leo. [23,24,25]. Moreover, in *P. capsici* heavy metal-limited hyphal growth was accompanied by reduced sporulation and even virulence manifested by the decreased expression of the laccase *PcLAC2* and necrosis-inducing NLP protein *PcNLP14* genes [25]. Thus, recognition of heavy metal impact on the biology and pathobiology of economically important phytopathogens seems to have a priority in the constantly progressing pollution of the environment.

One of the most dangerous oomycete plant pathogens on the global scale, generating huge losses in potato yield is *Phytophthora infestans* (Mont) de Bary [26]. As a causative agent of late blight it was responsible for the great Irish famine in the 19th century and even today this disease causes enormous yield losses estimated at USD 3 billion per year worldwide [26,27]. Importantly, *P. infestans* is capable of a very rapid evolution and adaptation to unfavorable environment conditions, causing inefficiency in the pathogen control [28]. Under natural conditions the pathogen survives winter in the soil as sexual oospores or as hyphae in infected plant debris, thus heavy metals deposited in the soil may affect *P. infestans* metabolism and its pathogenicity. It worth pointing that the natural Cd concentration in soil is relatively low, and soil contamination with Cd is mainly the result of human activities such as smelting, mining, electroplating [29], fossil fuel combustion, metallurgical works, sewage sludge, municipal and industrial wastes [30]. Unfortunately, there is no experimental knowledge concerning the oomycete response to the abiotic stress. The latest reports on *P. infestans* metabolism revealed RNS formation in the pathogen structures as an integral part of the pathogen biology and adaptation strategy to the host environment [31]. It should be noted that in filamentous fungi as microorganisms similar in morphology and habitat to the oomycetes, nitric oxide (NO) has also been implicated in abiotic stress responses. For example, mitochondrial NO production was observed in yeast cells exposed to anoxia [32]. In turn, enrichment of yeast cells with NO provided protection against oxidative stress provoked by heavy metals, heat shock stress, and high hydrostatic pressure [33,34]. In view of the above, the main aim of this study was to determine the effect of Cd, as a model heavy metal stress, on the nitro-oxidative status in *P. infestans* structures as well as the oomycete pathogenicity.

## 2. Results

### 2.1. Cadmium Stress Affects P. infestans in Vitro Growth and Sporulation

**Figure 1:**
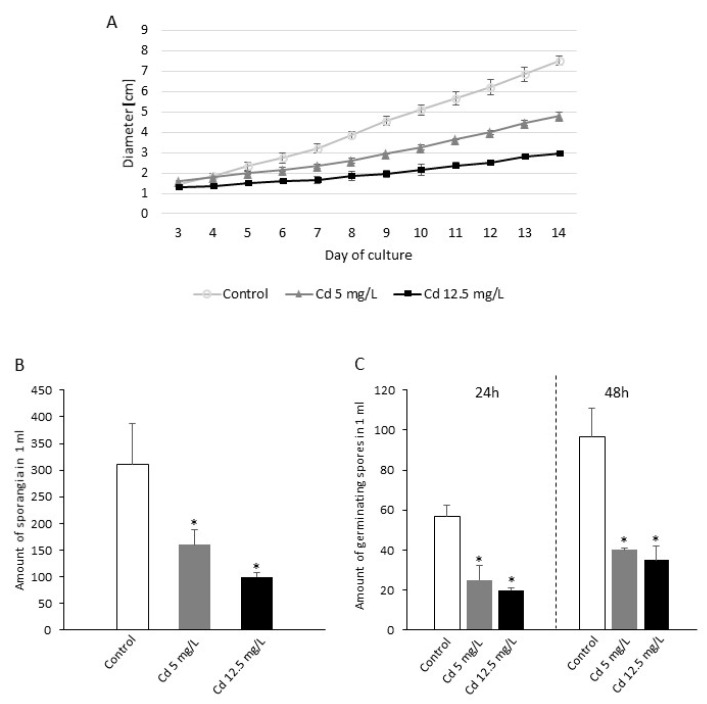
The effect of various Cd concentrations on *Phytophthora infestans in vitro* growth and sporulation. (**A**) Radial growth of *P. infestans* on medium supplemented with 0, 5, and 12.5 mg/L of Cd; (**B**) amount of sporangia formation in *P. infestans* culture treated with 0, 5, and 12.5 mg/L of Cd; (**C**) amount of germinating spores determined following a 24 and 48-h exposure to Cd stress. The results are an average from three independent experiments ± SD. Asterisks indicate values that differ significantly from the nontreated (control) *P. infestans* culture at *p* < 0.01 (*).

To assess a direct effect of Cd stress on *P. infestans in vitro* growth and sporulation, different concentrations of Cd supplied to the medium as CdCl_2_ were used (0–25 mg/L), allowing to determine the tolerance index of *P. infestans* to the heavy metal. The results showed that Cd significantly limited *in vitro* growth of the oomycete and the concentrations up to 5 mg/L of Cd included in the tolerance limit noted for *P. infestans* (Figure S1). Thus, 5 mg/L of Cd caused ca. 50% decrease of hyphal growth and reflected moderate heavy metal stress, whereas 12.5 mg/L of Cd caused ca. 75% decrease of hyphal growth area and was described as sublethal Cd stress (Figure 1A). Both Cd concentrations inhibited also sporangia formation and spore germination (Figure 1B,C). The amount of sporangia was reduced by 48% and 68% in response to 5 and 12.5 mg/L of Cd, respectively (Figure 1B). Additionally, both Cd doses delayed germination of spores to a similar degree: ca. 56% and 65% after 24 h, and 65% and 63% after 48 h of preparing the spore suspension from the culture growing under moderate and sublethal Cd stress, respectively (Figure 1C).

### 2.2. Cadmium Exposure Provokes Reactive Oxygen and Nitrogen Species Formation in P. infestans Structures

**Figure 2:**
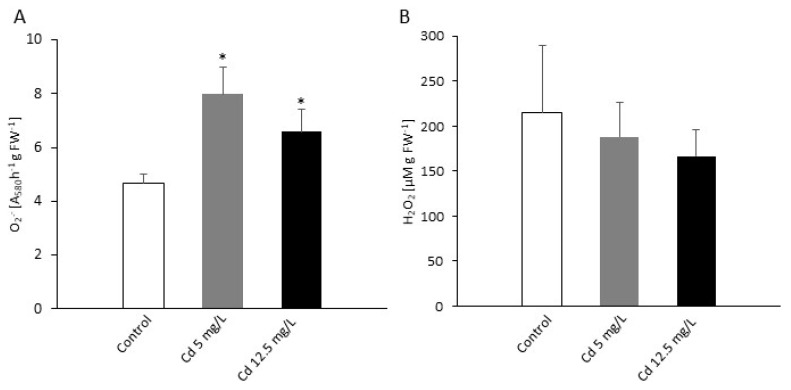
The effect of Cd on reactive oxygen species formation in *P. infestans*. (**A**) Superoxide radical and (**B**) hydrogen peroxide production at 14th day of the culture exposed to Cd stress. The results are an average from three independent experiments ± SD. Asterisks indicate values that differ significantly from the nontreated (control) *P. infestans* culture at *p* < 0.01 (*).

The observed inhibition of *P. infestans in vitro* growth was accompanied by marked changes in nitro-oxidative metabolism in *P. infestans* structures. Both Cd concentrations caused a significant increase in superoxide anion (O_2_¯) levels. The lower Cd dose caused a ca. 2-fold increase, while the higher one provoked only ca. 43% rise in O_2_¯ accumulation in comparison to the control culture of *P. infestans* (Figure 2A). Cadmium stress resulted also in a statistically insignificant drop in hydrogen peroxide (H_2_O_2_) formation (Figure 2B).

**Figure 3:**
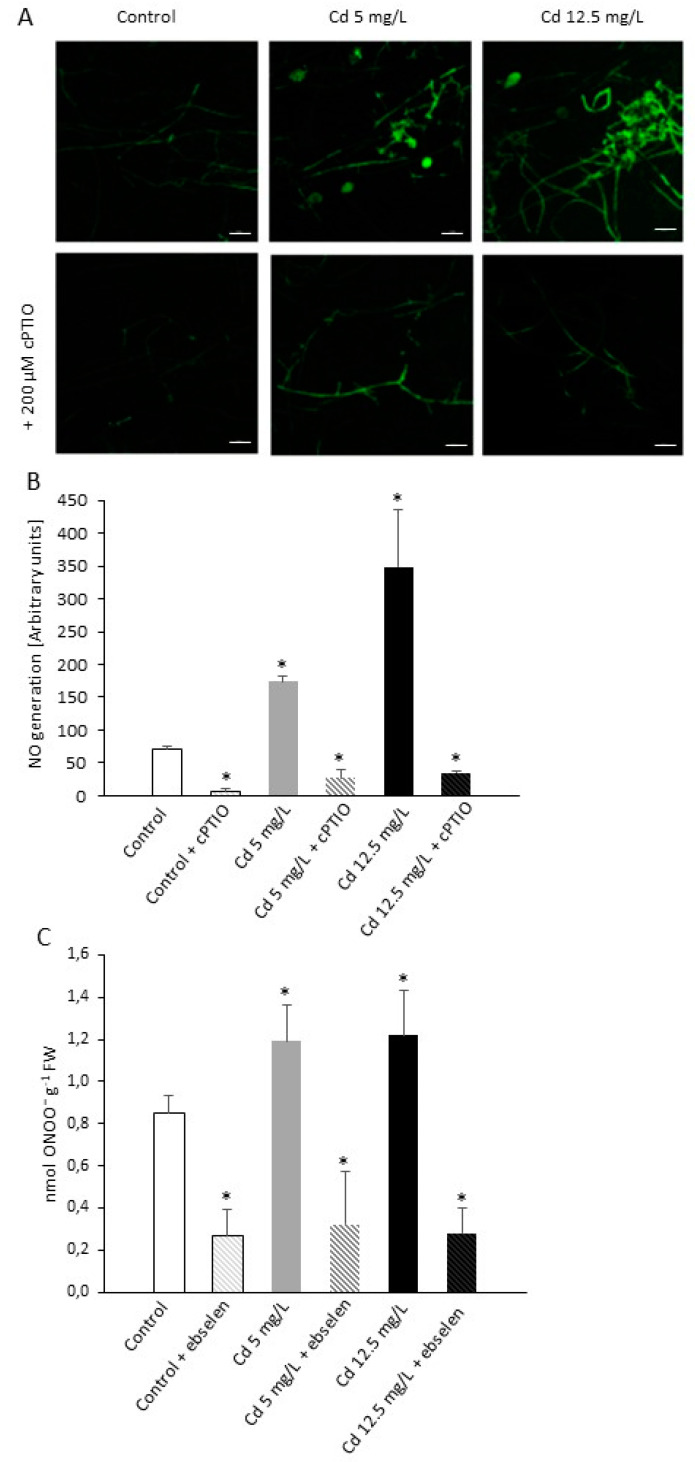
Bio-imaging of NO with DAF-2DA (4,5-diaminofluorescein diacetate) in *P. infestans* structures growing under Cd stress. (**A**,**B**) Nitric oxide was analyzed at 14th day of the culture growing in the presence of 0, 5, and 12.5 mg/L of Cd ± 200 µM cPTIO (2-phenyl-4,4,5,5,-tetramethylimidazoline-1-oxyl 3-oxide). Bars indicate 50 µm. Images show general phenomena representative of three independent experiments. (**C**) Peroxynitrite content in *P. infestans* at 14th day of the culture growing under Cd stress. The results are an average from three independent experiments ± SD. Asterisks indicate values that differ significantly from the nontreated (control) *P. infestans* culture at *p* < 0.01 (*).

Measurement of NO using a fluorescent indicator DAF-2DA (4,5-diaminofluorescein diacetate) revealed enhanced generation of this RNS in *P. infestans* structures growing *in vitro* in the presence of Cd. Hyphae exposure to the moderate Cd dose resulted in an over two-fold increase of NO production; in turn, sublethal heavy metal stress provoked a huge overproduction of NO localized in hyphae and sporangia of the pathogen. Moreover, application of cPTIO (2-phenyl-4,4,5,5,-tetramethylimidazoline-1-oxyl 3-oxide) as a specific NO scavenger resulted in a highly reduced NO-dependent fluorescence, confirming specificity of NO formation in *P. infestans* structures after Cd challenge (Figure 3A,B). Pathogen exposure to both Cd stresses triggered also a ca. 30% rise in peroxynitrite (ONOO¯) formation (Figure 3C).

### 2.3. Cadmium Affects the Nitro-Oxidative Pattern of Nucleic Acids and Proteins

**Figure 4:**
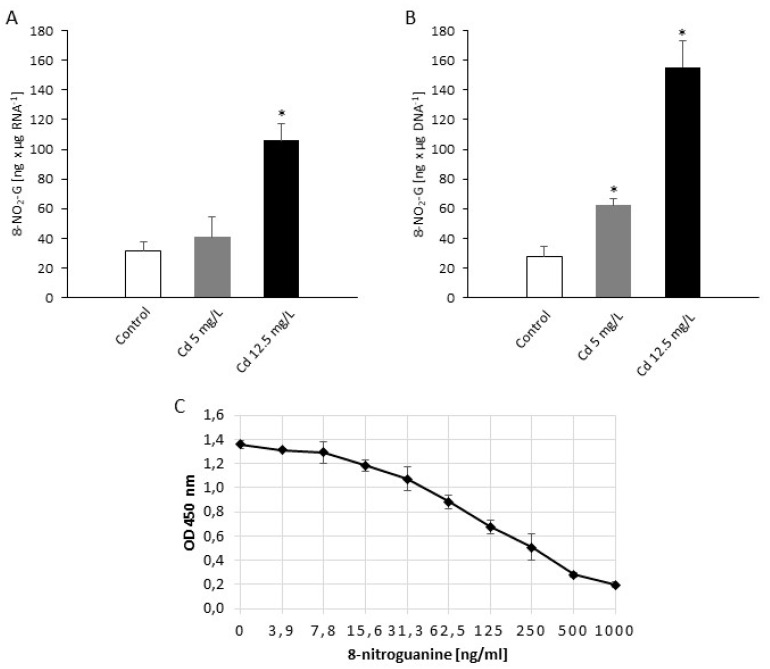
RNA and DNA nitration in *P. infestans* at 14th day of the culture exposed to Cd stress. (**A**) Quantification of nitrated RNA measured as 8-NO_2_-G content; (**B**) quantification of nitrated DNA measured as 8-NO_2_-G content; (**C**) 8-nitroguanine ELISA standard curve in the concentration range of 0–1000 ng/mL. The results are an average from three independent experiments ± SD. Asterisks indicate values that differ significantly from the nontreated (control) *P. infestans* culture at *p* < 0.01 (*).

To verify that Cd-mediated RNS formation is able to provoke nitration at the RNA and DNA levels in the *P. infestans* cellular environment, the accumulation of 8-NO_2_-G was monitored as a marker of nucleic acid nitration (Figure 4). As had been expected, the heavy metal presence resulted in 8-NO_2_-G overaccumulation within both the RNA and DNA pools. However, only a sublethal Cd dose provoked a statistically significant, ca. 4-fold increase of 8-NO_2_-G RNA accumulation in relation to the control (Figure 4A). A similar, Cd dose-dependent trend was observed in nitrated DNA. Briefly, a definitely higher level, ca. 6-fold rise of 8-NO_2_-G DNA was noted in pathogen structures exposed to sublethal Cd stress (Figure 4B).

**Figure 5:**
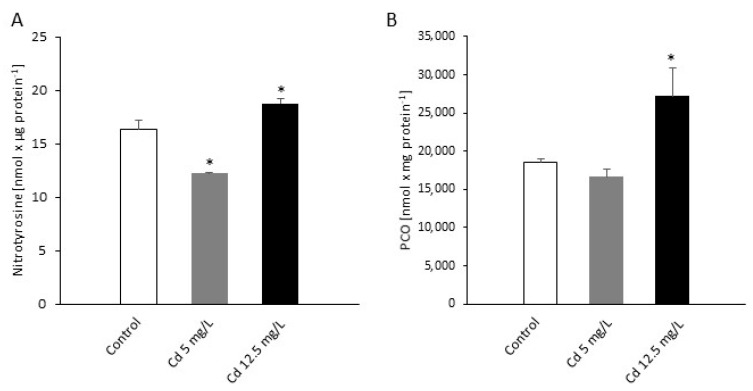
Protein nitration and carbonylation in *P. infestans* at 14th day of the culture exposed to Cd stress. (**A**) Quantification of nitrated proteins measured as nitrotyrosine content; (**B**) content of carbonylated proteins. The results are an average from three independent experiments ± SD. Asterisks indicate values that differ significantly from the nontreated (control) *P. infestans* culture at *p* < 0.01 (*).

An opposite effect was observed in relation to the protein pool undergoing nitration and oxidation as a consequence of Cd-induced RNS/ROS formation. A total amount of nitrated proteins was ca. 25% lower in *P. infestans* structures growing under moderate Cd stress; in turn, the higher Cd concentration provoked only ca. 15% increase in the pool of nitrated proteins when compared to the control (Figure 5A). A significant difference between moderate and sublethal Cd stresses was noted also in the case of protein carbonylation. While at the lower Cd concentration a slight decrease (ca. 10%) in protein carbonylation was noted, the higher metal dose increased this type of protein oxidation by as much as over 30% (Figure 5B).

### 2.4. Cadmium Accelerates P. infestans Antioxidative Response

**Figure 6:**
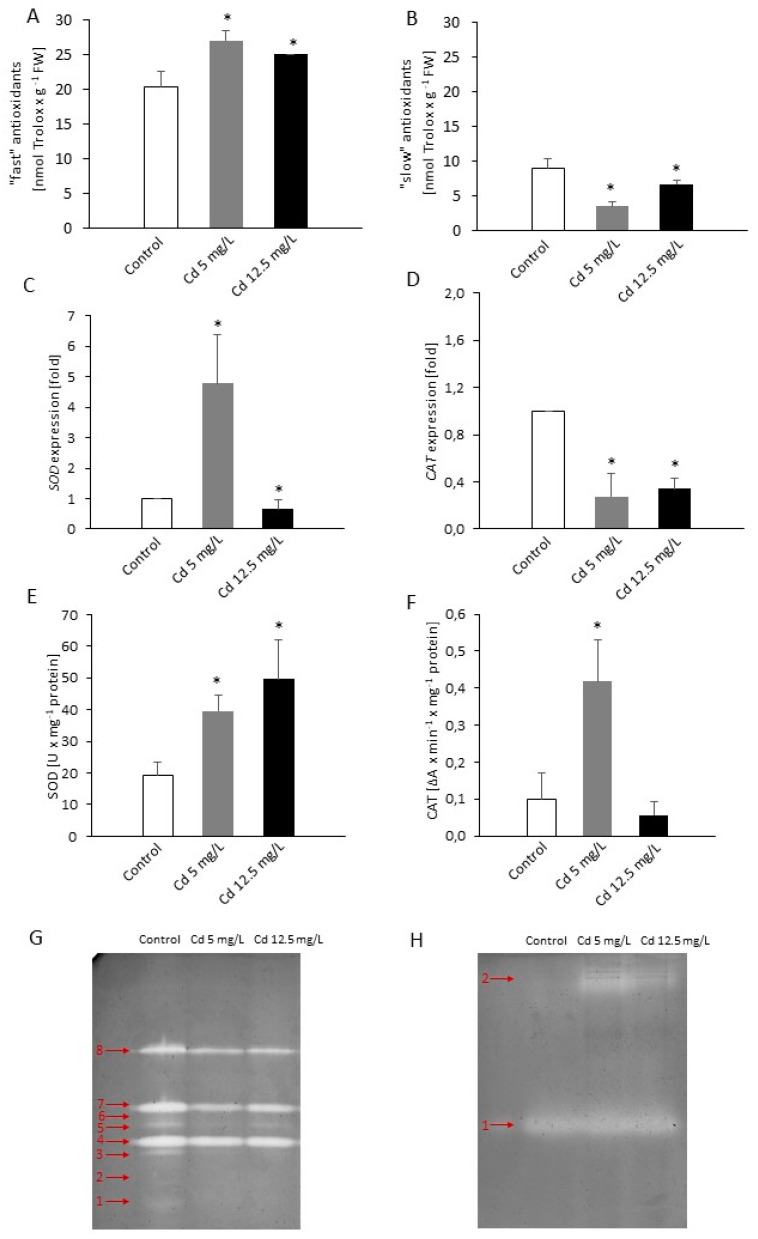
The effect of Cd on the selected elements of the antioxidant system in *P. infestans*. A total antioxidant capacity of *P. infestans*: (**A**) the content of “fast” and (**B**) “slow” antioxidants; RT-qPCR analysis of (**C**) *SOD* (superoxide dismutase) and (**D**) *CAT* (catalase) genes expression; the activity of (**E**) SOD and (**F**) CAT. The results are an average from three independent experiments ± SD; asterisks indicate values that differ significantly from the nontreated (control) *P. infestans* culture at *p* < 0.01 (*). Representative images of isoenzyme patterns of (**G**) SOD and (**H**) CAT in *P. infestans* structures at 14th day of the culture growing under Cd stress.

To determine the effect of Cd stress on the vr *P. infestans* antioxidant system, total antioxidative capacity (TAC), transcript accumulation, and biological activity of the key antioxidant enzymes, i.e., catalase (CAT) and superoxide dismutase (SOD), were evaluated (Figure 6).

The Cd provoked changes in ROS/RNS metabolism in *P. infestans* structures were accompanied by an enhanced content of the so-called fast antioxidants, including the reduced pool of ascorbate and glutathione; however, the highest level was noted under moderate Cd stress conditions (Figure 6A). An opposite effect was observed in the case of the so-called slow antioxidants involving e.g., residues of tyrosine and tryptophan in proteins; briefly, the pool of these antioxidants decreased by ca. 60% and 30% in response to moderate and sublethal Cd dose exposure, respectively (Figure 6B).

The expression analysis of the genes encoding selected antioxidant enzymes clearly indicated that moderate Cd stress effectively increased transcription of SOD1, which product is responsible for scavenging the superoxide anion radical (Figure S2, Figure 6C). RT-qPCR assays of *SOD1* showed that 5 mg/L of Cd provoked a ca. 5-fold increase of the gene expression. An opposite effect was observed at the highest Cd concentration (12.5 mg/L), since a decreased *SOD1* expression in comparison to the control was noted. The activity of SOD was accelerated at both Cd concentrations. Although a ca. 2-fold and 2.5-fold rise of the enzyme activity was found in response to moderate and sublethal Cd doses, respectively (Figure 6E), the SOD isoenzyme pattern detected in *P. infestans* growing under Cd stress was reduced from 8 to 5 protein bands described as nos. 3, 4, 5, 7, and 8 (Figure 6G, Figure S3).

Although heavy metal stress significantly diminished CAT gene expression (Figure 6D), an over 4-fold increase of CAT activity was observed in vr *P. infestans* growing at the lower Cd concentration (Figure 6F). Simultaneously, two CAT isoforms appeared in response to Cd stress; however, the Cd-responsive CAT protein band (no. 1) was more visible in extracts obtained from vr *P. infestans* structures exposed to moderate Cd stress (Figure 6H, Figure S4).

### 2.5. Moderate Cadmium Stress Amplifies P. infestans Pathogenicity on Potato

**Figure 7:**
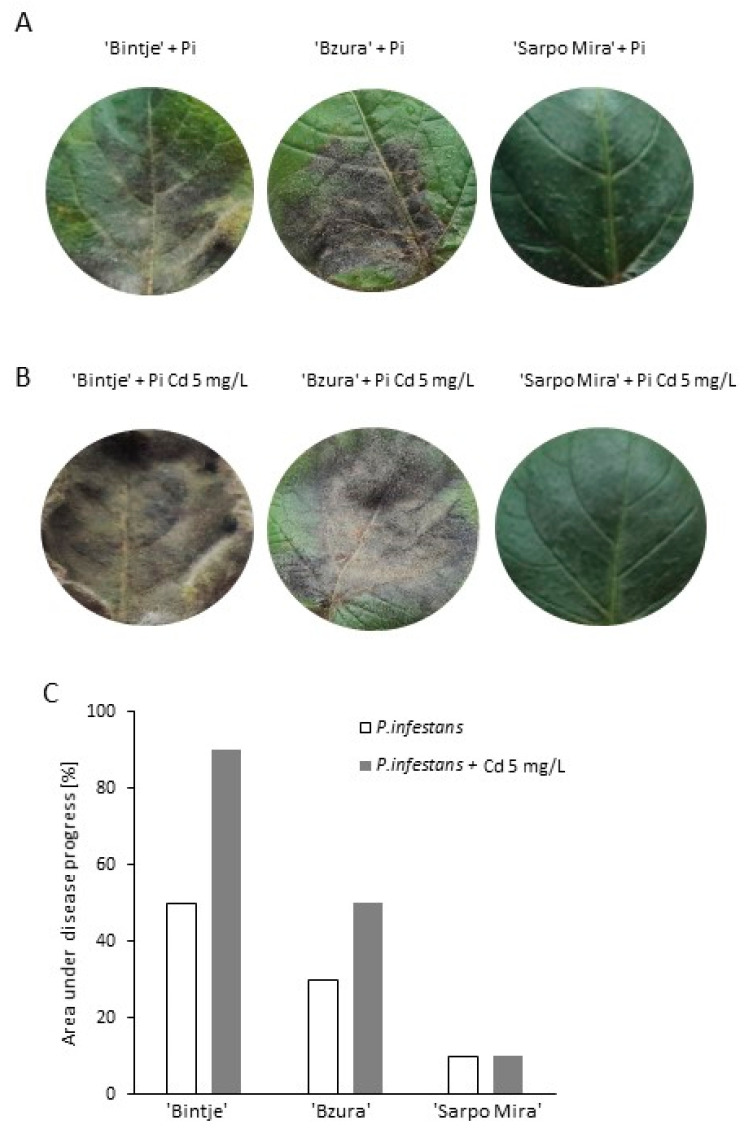
The effect of moderate cadmium stress on *P. infestans* aggressiveness. Representative images of leaves of three potato cultivars inoculated with vr *P. infestans* (**A**) growing in the absence of Cd (control media) and (**B**) in the media supplemented with Cd 5 mg/L; (**C**) the comparison of late blight disease development on the leaves of three potato cultivars inoculated with vr *P. infestans* growing on a medium without and with Cd 5 mg/L.

To assess if Cd stress affects vr *P. infestans* aggressiveness, potato leaves were inoculated with the spore suspension prepared from hyphae growing under moderate Cd stress (5 mg/L). For the experiment three cultivars of potato were used, i.e., Bintje (Bi), Bzura (Bz), and Sarpo Mira (SM). The highest infection rate after inoculation with vr *P. infestans* growing on the control medium showed cv. Bintje, since disease spots covered 50% of Bintje leaf area at 7th day post inoculation (dpi). In turn, cv. Bzura and Sarpo Mira exhibited disease symptoms on 30% and 10% of leaf area, respectively (Figure 7A). When the used potato cultivars were inoculated with vr *P. infestans* spore suspension prepared from the culture growing under moderate Cd stress, significant changes in the rate of late blight development were observed. Disease symptoms at 7th dpi covered a much larger area of the leaves in cultivars Bintje and Bzura when compared to leaves inoculated with *P. infestans* growing under the control conditions (Figure 7B). Based on the disease index, inoculation with the Cd-stressed pathogen provoked late blight symptoms covering ca. 90% of ‘Bintje‘ leaf area, 50% of ‘Bzura‘ leaf area, and 10% of ‘Sarpo Mira‘ leaf area, respectively (Figure 7C).

**Figure 8:**
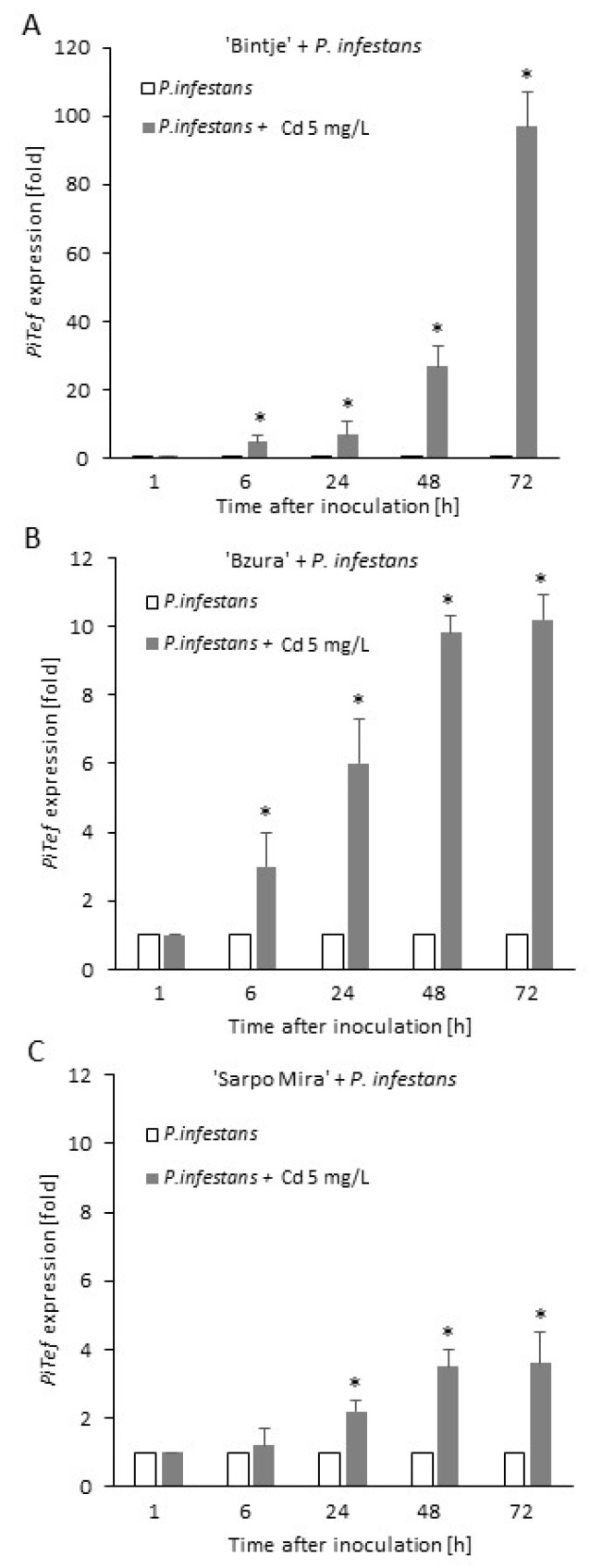
Expression analysis of the *Phytophthora infestans* Tef1 gene (*PiTef1*) in leaves of three potato cultivas (**A**) Bintje, (**B**) Bzura, (**C**) Sarpo Mira inoculated with vr *P. infestans* growing in the absence of Cd (control media) and in the media supplemented with Cd 5 mg/L. The values are normalized to the potato Ef1-α gene. The results are an average from three independent experiments ± SD. Asterisks indicate values that differ significantly from the nontreated *P. infestans* inoculated leaves at *p* < 0.01 (*).

The rate of *P. infestans* infection was also quantified by RT-qPCR. The transcript level of the *Pitef1* gene was calculated relative to the expression level of the potato *Ef1-α* gene according to Orłowska et al. [35]. In general, addition of Cd (5 mg/L) to the growing medium of vr *P. infestans* provoked an enhanced expression of *Ptef1* under *in planta* conditions in comparison to leaf inoculation with vr *P. infestans* growing in the control medium (Figure 8). The *Pitef1* gene expression started to increase from the 6th hour post inoculation (hpi) in Bi leaves and the highest transcript accumulation, i.e., an over 95-fold rise, was noted at 72 hpi (Figure 8A). A similar Cd-mediated effect was observed during the Bz-vr *P. infestans* interaction and a ca. 10-fold increase of the transcript accumulation was noted at 48 and 72 hpi (Figure 8B). Cadmium stress elevated expression of *Pitef1 in planta* also during the pathogen interaction with the SM genotype. The gene expression started to increase from 24 hpi in SM leaves and an over 3-fold increase was noted at 48 and 72 hpi (Figure 8C). 

## 3. Discussion

No comprehensive studies are available on the effects of nonessential toxic metals on biology and pathobiology of plant pathogens. However, there is a growing body of evidence suggesting a link between heavy metal stress and modification of microorganism pathogenicity [36,37].

The presented study clearly indicates that Cd stress affects *in vitro* growth of the oomycete plant pathogen *P. infestans*. The higher the metal concentration applied, the greater reduction was observed in radial growth of vr MP 977 *P. infestans* hyphae. The most adverse effect was provoked by Cd at the concentration of 12.5 mg/L reflecting the sublethal Cd dose. It was earlier found that some plant pathogens belonging to oomycetes, e.g., *Pythium debaryanum*, can exhibit a high degree of tolerance to heavy metals, since it can grow in the presence of high metal doses including 100 mg/L of Cd, Pb, Cu, and Zn. What is more, a low Pb concentration (3 mg/L) even stimulated the growth rate of *P. debaryanum* [19]. Lorenzo-Gutiérrez et al. [38] underlined that some soil microorganisms may exhibit a high tolerance to heavy metals, probably acquired through their evolutionary adaptation to contaminated environments. Therefore, the survival of fungal or fungal-like cultures should not be surprising, even at metal doses that are lethal for organisms belonging to distant systematic groups.

In general, heavy metals can affect morphogenesis of the vegetative hyphae or mycelium as well as sexual and asexual reproduction. The Cd-reduced growth of vr MP 977 *P. infestans* culture was accompanied by low sporangia abundance with a delayed germination rate. A similar effect was observed earlier in the oomycete *Dictyuchus carpophorus* treated even with low concentrations of Cd [24]. Heavy metals at concentrations of 0.5–1.0 mM Cu, Cr, and Hg were also able to inhibit hyphae growth and sporulation in the oomycete *Phytophthora capsici* [25]. According to Gill [39] the reduced growth rate of pathogen hyphae in the presence of Cd may be a result of Cd interfering with the uptake, transport, and use of several elements (Ca, Mg, P, and K) as well as water by the microorganism.

It is well known that even low concentrations of Cd as an environmental pollutant provoke cell redox misbalance manifested by ROS/RNS overproduction, which may contribute to the metal toxicity [25]. As earlier documented, *in vitro* growth of the vr MP 977 *P. infestans* is accompanied by RNS formation and it is accelerated during *in planta* sporangia development [31]. In the present study we found that Cd stress boosted NO formation in both sporangia and hyphae under *in vitro* conditions. Since RNS could mediate nuclear division or degeneration of a proportion of the nuclei in the sporangium providing sporangia with the ability to release zoospores in rapid succession [31], the sporangia-localized NO overproduction in response to heavy metal stress seems to be essential for pathogen survival. Moreover, NO can accelerate formation and maturation of asexual reproductive structures by controlling the expression of the conidiation related con genes [40].

It is important to note that NO overproduction noted in *P. infestans* structures growing under Cd stress was orchestrated with superoxide elevation, favoring a microenvironment for ONOO¯ formation. However, both moderate and sublethal stresses boosted ONOO¯ formation in the same extent. Further insight into the cellular redox status of *P. infestans* exposed to heavy metal stress revealed that RNS overproduction is accompanied by nitro-oxidative modifications of the key biomolecules including proteins and nucleic acids. Although protein nitration is still perceived as a marker of nitro-oxidative stress, our results suggest that this modification acts as a physiological regulator to dynamically redirected cellular metabolism under environmental disorders. Moderate Cd stress diminished the pool of nitrated proteins, whereas the sublethal one only slightly increased the nitrated protein level in comparison to the control. A slight fluctuation within the nitrated protein pool was also observed earlier in vr MP 977 *P. infestans* structures during *in planta* growth despite the host’s nitro-oxidative environment favoring biomolecule modification [31]. Thus, changes in the protein pool undergoing Tyr nitration phenomena in *P. infestans* may reflect a homeostasis misbalance connected with the adaptation strategy of the microorganism to different microenvironments, including the host or heavy metal contamination.

Cadmium-provoked nitrative modification in vr MP 977 *P. infestans* structures involved also nucleotides in DNA and RNA. The formation of 8-nitroguanine occurred to a much greater extent under the sublethal Cd dose, indicating that these DNA/RNA lesions can contribute to the metal toxicity. Although this statement is in line with human/animal models where the occurrence of nitrated nucleic acids has been regarded as a marker of pathogenesis and damage, recent findings in plant models suggest that the nitrative modification of RNA may function as a smart redox switch of gene expression [41]. A functional consequence of posttranscriptional mRNA modifications consists in hampered translation, resulting in a decreased level of encoded proteins or even expanding the diversity of proteins through recoding [42]. Importantly, quantification of nitrated RNA measured as the 8-NO_2_-G content in vr MP 977 *P. infestans* showed no statistically significant increase of this modification at moderate Cd stress, suggesting a scenario that RNA nitrative modification might function as a swift adjustment of *P. infestans* metabolism under abiotic stimuli rather than merely nitrative damage of RNA. Certainly future identification of mRNA nitration targets will provide a verification of these assumptions.

To cope with metal toxicity, microorganisms activate antioxidant systems [43]. In phytopathogens induction of antioxidant enzymes in response to the heavy metal mediated oxidative stress has been demonstrated in a fungus *P. chrysosporium* [44] and an oomycete *Phytophthora capsici* [25]. The antioxidant response of *P. infestans* engaged transcriptional, post-translation, and enzymatic machinery, which facilitates adaptation to the nitro-oxidative environment provoked by Cd. Although a number of genes whose products are potentially involved in maintaining cell redox balance (*SOD1, PiCAT5, CATG, GPX2, PiPRX2*, and *PiTPX2*) have been studied in vr MP 977 *P. infestans*, only *SOD1*, *GPX2*, and *PiTPX2* were dose-dependently upregulated by Cd in the pathogen structures (Figure 6C, Figure S2). The obtained results clearly showed that the oomycete exposed to moderate Cd stress activates primarily SOD at both transcript and enzyme activity levels to cope with the Cd-induced oxidative stress; however, native electrophoresis revealed that Cd stress affects some SOD isoforms. These results are in line with the study of Ighodaro and Akinloye [45] indicating SOD as the first detoxification enzyme and the most powerful antioxidant within the cell exposed to ROS. Moreover, a high SOD expression at transcript and activity levels in vr MP 977 *P. infestans* may balance ONOO¯ formation directly responsible for nitrative biomolecule modification.

Catalase may also play an important role in adaptation and ultimate survival of *P. infestans* during periods of stress and stress-induced ROS over-accumulation. In confirmation, the Cd-responsive CAT isoform correlated with an enhanced enzyme activity was detected only in *P. infestans* exposed to moderate Cd dose. A similar effect was observed in a fungus *Phanerochaete chrysosporium*, in which a lower Cd concentration or short-term Cd stress was effective in induction of CAT activity; in turn, larges Cd doses or longer stress exposure did not provoke the enzyme activity [46]. Both CAT and SOD activities were also elevated in yeast *Trichosporon cutaneum* treated with Cd and Cu and were indicated as key enzymes engaged in ROS scavenging in the fungus [22].

An ROS/RNS imbalance and enhanced antioxidant response noted in *P. infestans* structures growing under moderate Cd stress was also associated with an enhanced aggressiveness of the pathogen; however, it may be a metal dose-dependent phenomenon. Inoculation of potato leaves with the spore suspension prepared from vr *P. infestans* MP 977 culture growing in media supplemented with Cd (5 mg/L) provoked accelerated disease symptoms on two potato cultivars, i.e., Bi and Bz. The rate of disease development measured as the disease index was not affected in the case of SM; however, molecular assessment of disease progress revealed a significantly higher *Pitef* gene expression under *in planta* condition starting from the first day after SM inoculation. These results clearly show that in all the analyzed interactions the rate of infection development was accelerated when spores were derived from the Cd stressed vr *P. infestans* culture. A well-known defense strategy of host organisms is to produce ROS to combat the pathogen. The ROS burst has been detected in many plant-pathogen interactions as an early event of the plant defence strategy, including the potato–*P. infestans* interaction [47]. Thus, an enhanced aggressiveness of *P. infestans* growing in the presence of Cd might be linked to the efficient elimination of host (potato)-derived ROS despite the fact that the Cd dose delayed germination of spores. More precisely, an enhanced aggressiveness of *P. infestans* may result from the Cd-activated antioxidant system, i.e., SOD and CAT. Notably, cytosolic Cu/Zn Sod1 was documented as a virulence factor for *Cryptococcus neoformans* and *Candida albicans*. In turn, in a human pathogen *Aspergillus fumigatus* all SODs were required for full virulence of the pathogen [48]. Interestingly, the heavy metal-induced expression of *PcaA*, a cation ATPase, was proved to provide Cd tolerance in *A. fumigatus* and support its virulence in the *Galleria mellonella* model [34]. As the authors stated, an increased PcaA protein level favors ROS detoxification in *A. fumigatus* protecting the microorganism against both Cd-mediated and host-derived oxidative stress. Importantly, the protection capacity of Cd-induced metabolic events to subsequent stress was demonstrated earlier in plants. As documented Stroiński et al. [49,50] Cd was able to induce cross-resistance phenomenon of potato to *P. infestans* since tubers and leaves of susceptible potato cv. Bi exposed to the heavy metal showed induction of basal defense and reduced symptoms of late blight disease.

Concluding, Cd stress not only inhibited cellular growth and caused biochemical changes in vr *P. infestans*, but it also favored pathogenicity of the oomycete. The presented results shed new light on the mechanism, showing that the nitro-oxidative homeostasis imbalance underlies a link between heavy metal stress and modification of microorganism pathogenicity. However, whether the nature of the observed phenomenon of an enhanced aggressiveness of *P. infestans* underlies stress cross-protection, observed earlier in yeast cells [51] and/or involves epigenetic mechanisms and transcriptional reprogramming of pathogenicity related genes will require future experimental verification.

## 4. Materials and Methods

### 4.1. Pathogen Culture

*Phytophthora infestans*, a virulent race 1.2.3.4.6.7.10 (MP 977) was supplied by the Plant Breeding and Acclimatization Institute, (IHAR) Research Division at Młochów, Poland. The pathogen was cultured for 14 days at 16 °C in the dark on a cereal-potato medium prepared by single components with the addition of dextrose (Sigma; Saint-Louis, MO, USA). For *in planta* experiments potato leaves were inoculated by spraying with the zoospore suspension at a concentration of 2.5 × 10^5^ per 1 mL of water as described by Arasimowicz-Jelonek et al. [52] and then leaves were kept at 16 °C and 95% humidity.

### 4.2. Plant Material

Plants of three potato cultivars Bintje, Bzura and Sarpo Mira came from the Potato Gene Bank (Plant Breeding and Acclimatization Institute—IHAR in Bonin, Bonin, Poland). Potato seedlings propagated through *in vitro* culture were transferred to the soil and kept in a phytochamber with 16 h of light (180 μmol·m^−2^·s^−1^) at 18 ± 2 °C and 40% humidity up to the stage of 10 leaves.

### 4.3. Assessment of the Area under Disease Progress

In order to estimate the development of disease symptoms, percentage of the leaf area under disease progress (*n* = 15) was evaluated after 7 days from spraying the leaves with a *P. infestans* spore suspension (2.5 × 10^5^ per 1 mL) prepared from hyphae growing in the absence of Cd (control media) and the media supplemented with Cd 5 mg/L.

### 4.4. Cadmium Stress, Hyphal Growth, and Sporulation of P. infestans

The heavy metal (Cd^2+^) stock solution was obtained from its chlorine salt (P.O.Ch.; Gliwice, Poland; grade min. 99.5%) that was dissolved in sterilized water. For *in vitro* growth experiments the stock solution was added to the growing medium to reach the following concentrations of Cd: 0, 1, 2.5, 5, 12.5, and 25 mg/L. Two Cd concentrations, i.e., 5 and 12.5 mg/L reflecting moderate and sublethal heavy metal stress, respectively, were selected for further analyses. Material for molecular and biochemical analyses was collected on the 14th day of the culture.

Radial growth of *P. infestans* was measured every day for a period of 14 days—the time of pathogen culture. The spore suspension for the calculation of sporangia and germinating spore counts was freshly prepared from the control or Cd-exposed hyphae and the number of sporangia were determined using the Bürker chamber. To assess the number of germinating spores, microscopic counting was performed using 1% agar.

### 4.5. Superoxide Radical Production

Superoxide radical anion (O_2_¯) detection was performed using the method with nitroblue tetrazolium (NBT) described by Doke [53]. Briefly, hyphae (0.05 g) of *P. infestans* were incubated in 3 mL of incubation mixture (0.05% NBT (SERVA; Heidelberg, Germany), 0.1 mM EDTA (BioShop; Mainway, Burlington, ON, Canada), 0.065% sodium azide (P.O.Ch.; Gliwice, Poland), and 50 mM potassium phosphate buffer, pH 7.8 (Chempur; Piekary Śląskie, Poland). The incubation mixture without the tested material was used as a blank sample. Samples were incubated in the dark at room temperature (RT) for 1 h. After incubation 1.8 mL of the reaction mixture were heated at 85 °C for 15 min to complete the reaction. After cooling on ice absorbance at a wavelength of 580 nm was determined by measuring the amount of reduced NBT to diformazane. Superoxide generation was expressed as the change in absorbance A580 per 1 g fresh weight (FW) per 1 h.

### 4.6. Hydrogen Peroxide Accumulation

Hydrogen peroxide (H_2_O_2_) concentration was assayed spectrophotometrically using the titanium (Ti^4+^) method described by Becana et al. [54]. Hyphae (0.1 g) of *P. infestans* were homogenized in 1.6 mL of 0.1 M potassium phosphate buffer (pH 7.8) (Chempur; Piekary Śląskie, Poland). After centrifugation (13,000 *g* for 25 min at 4 °C) the supernatant was collected and used for assays. The reaction mixture (1.5 mL) contained 0.1 M potassium phosphate buffer (pH 7.8) (600 µL), enzymatic extract (400 μL), and titanium reagent (500 µL). Titanium reagent was prepared on the day of the assay by mixing 0.6 mM solution of 4-(2-pyridylazo) resorcinol (Sigma; Saint-Louis, MO, USA) and 0.6 mM potassium titanium tartrate (Sigma; Saint-Louis, MO, USA) at a 1:1 ratio. The concentration of H_2_O_2_ was determined by measuring absorbance at a wavelength of 508 nm against a calibration curve and expressed as µmol H_2_O_2_ per 1 g FW.

### 4.7. Bio-Imaging of Nitric Oxide

Nitric oxide *in vivo* formation was detected using DAF-2DA (4,5-diaminofluorescein diacetate) fluorochrome (Calbiochem; San Diego, California, U.S; excitation 495 nm; emission 515 nm). Briefly, *P. infestans* hyphae were incubated in the dark for 1 h in 10 mM Tris-HCl buffer (pH 7.4) (Bio-Rad; Hercules, CA, USA) containing 10 µM DAF-2DA. After incubation the mycelium was washed twice in 10 mM Tris-HCl buffer (pH 7.4) (Bio-Rad). Samples were examined under a Zeiss LSM 510 confocal microscope (Carl Zeiss, Jena, Germany) equipped with standard filters and collection modalities for DAF-2DA green fluorescence. Images were processed and analyzed using the ImageJ software.

### 4.8. Peroxynitrite Detection

The level of peroxynitrite was assayed using folic acid as the peroxynitrite scavenger, giving high fluorescent emission products [55]. Hyphae (0.5 g) were immersed in the incubation mixture, containing barbital buffer solution (pH 9.4) (Warchem; Marki, Poland) and folic acid (1.0 × 10^−5^ mol L^−1^) (Merck; Kenilworth, NJ, USA). Fluorescence intensity of the solution was recorded at 460 nm with the excitation wavelength set at 380 nm. The standard curve was prepared for 3-Morpholinosydnonimine (SIN-1) from Calbiochem as a donor of peroxynitrite at the range of concentration from 1 to 14 nM.

### 4.9. Protein 3-Nitrotyrosine Assay

Hyphae of *P. infestans* (0.2 g) were ground in liquid nitrogen to a fine powder, then it was suspended in buffer containing 50 mM Tris–HCl (pH 7.6) (Bio-Rad; Hercules, CA, USA) with 2 mM EDTA (BioShop), 2 mM DTT (Sigma; Saint-Louis, MO, USA), and 1 mM PMSF (Sigma; Saint-Louis, MO, USA). After centrifugation (10,000 *g* for 15 min at 4 °C) the supernatant was collected and the protein concentration was determined with the Bradford [56] assay. 3-nitrotyrosine in a protein sample was measured using the OxiSelect^TM^ Nitrotyrosine ELISA Kit (Cell Biolabs; San Diego, CA, USA; STA-305) according to the manufacturer’s protocol. Optical density was measured at 450 nm using an IMARK^TM^ Microplate Reader (Bio-Rad; Hercules, CA, USA). The 3-nitrotyrosine content in protein samples was determined by comparing with the predetermined 3-nitrotyrosine standard curve. Each sample was analyzed in triplicate on ELISA microplates.

### 4.10. Protein Carbonylation Assay

The level of carbonylated proteins (PCO) was evaluated acording to Colombo et al. [57]. Briefly, *P. infestans* protein samples at concentration of 1 mg/mL were incubated in the dark for 1 h with 2,4-dinitrophenylhydrazine (DNPH) (Sigma; Saint-Louis, MO, USA) solution. Then, 1.2 mL of 20% TCA (Sigma; Saint-Louis, MO, USA) was added to protein samples and incubated on ice for 15 min. After centrifugation (10,000 *g* for 5 min at 4 °C), protein pellet was washed twice with 1 mL of 20% TCA. Next, the pellet was washed with 1 mL of ethanol:ethyl acetate (1:1 *v*/*v*) solution (P.O.Ch.; Gliwice, Poland) in order to remove any free DNPH, and then samples were centrifuged (10,000 *g* for 10 min at 4 °C). The pellet was dried out and next resuspended in 1 mL of 6 M guanidine hydrochloride (Sigma; Saint-Louis, MO, USA) at 37 °C for 15 min. The absorbance was measured at 366 nm. The calculation of PCO content was based on the fact that the molar extinction coefficient (ε) for DNPH at 375 nm is 22,000 M^−1^ cm^−1^.

### 4.11. RNA Extraction

Hyphae of *P. infestans* were frozen in liquid nitrogen and stored at 80 °C until use. For RNA extraction hyphae (0.150 g) were ground to a fine powder and total RNA was extracted using TriReagent (Sigma; Saint-Louis, MO, USA) according to the manufacturer’s instructions.

### 4.12. DNA Extraction

Hyphae of *P. infestans* were frozen in liquid nitrogen and stored at 80 °C until use. For DNA extraction hyphae (0.150 g) were ground to a fine powder and then homogenized in buffer containing 200 mM Tris-HCl (pH 7.5) (Bio-Rad; Hercules, CA, USA), 250 mM NaCl (Stanlab; Lublin, Poland), 25 mM EDTA (BioShop; Mainway, Burlington, ON, Canada), 10% SDS (Sigma; Saint-Louis, MO, USA), and RNase A (ThermoFisher; Waltham, MA, USA) was added to each sample. After incubation (30 min at RT with mixing) the phenol-chloroform-isopropanol mixture (1:2:1) (BioShop; Mainway, Burlington, ON, Canada) was added and samples were mixed. After centrifugation (10,000 *g* for 12 min at 4 °C) the upper layer was collected, mixed with chloroform, and centrifuged (10,000 *g* for 12 min at 4 °C). Then the upper layer was mixed with isopropanol, incubated for 10 min at RT, and centrifuged (10,000 *g* for 12 min at 4 °C). The supernatant was removed, the precipitate was air dried, and dissolved in H_2_O_DEPC_.

### 4.13. 8-NO_2_-G Quantification

The level of 8-nitroguanine was quantified with the OxiSelect^TM^ Nitrosative DNA/RNA Damage ELISA Kit (Cell Biolabs; San Diego, CA, USA; STA-825). For the analysis 10 µg of total RNA or DNA were used. Further procedures were carried out according to the manufacturer’s instructions. The absorbance of the samples was measured at a wavelength of 450 nm using an IMARK^TM^ Microplate Reader (Bio-Rad; Hercules, CA, USA). The 8-NO_2_-G content was determined by comparing with the predetermined 8-NO_2_-G standard curve. Each sample was analyzed in triplicate on ELISA microplates.

### 4.14. Total Antioxidative Capacity

Total antioxidative capacity (TAC) was determined based on the ability of the antioxidants present in the extract to reduce the 2,2′ azinobis-(3-ethylbenzothiazoline-6-sulfonic acid) (ABTS) cation radical according to Bartosz [58]. The initial ABTS^+^ solution was diluted with 0.1 M potassium phosphate buffer (pH 7.4) (Chempur; Piekary Śląskie, Poland) to set absorbance at a wavelength of 414 nm on 1.0. Hyphae (0.1 g) were homogenized in 0.8 mL of 5% TCA (Sigma; Saint-Louis, MO, USA). After centrifugation (15,000 *g* for 30 min at 4 °C) the supernatant was collected and used for assays. The volume of 980 µL diluted ABTS^+^ was added to a cuvette and absorbance (A0) at a wavelength of 414 nm was measured. Then, 20 µL of the extract was added and absorbance was measured again after 10 s (A2) and 30 min (A1), respectively. Fast antioxidants were calculated as ΔA_fast_ = A1 − A0, whereas slow antioxidants were calculated as ΔA_slow_ = (A2 − A1) − (A2’ − A1’). The calibration curve was prepared by successively adding 5 μL portions of 0.01 mM Trolox^®^ (Sigma; Saint-Louis, MO, USA) to ABTS^+^ and measuring a decrease of absorbance. The final result of total antioxidative capacity was expressed in mM Trolox × g^−1^ FW.

### 4.15. Superoxide Dismutase Activity

Superoxide dismutase was assayed by measuring SOD ability to inhibit the photochemical reduction of NBT using the method of Beauchamp and Fridovich [59]. Fresh hyphae (0.05 g) were homogenized in 0.05 M sodium-phosphate buffer (pH 7.0) (Chempur; Piekary Śląskie, Poland), containing 1 mM EDTA (BioShop; Mainway, Burlington, ON, Canada), 1% PVPP (Sigma; Saint-Louis, MO, USA), and 0.01 M NaCl (Stanlab; Lublin, Poland). After centrifugation (15,000 *g* for 30 min at 4 °C) the supernatant was collected and used for assays. The assay mixture contained 0.05 M sodium phosphate buffer (pH 7.8) (Chempur; Piekary Śląskie, Poland), 13 mM methionine (Sigma; Saint-Louis, MO, USA), 75 μM NBT (SERVA; Heidelberg, Germany), enzymatic extract, and 2 μM of riboflavin (SERVA; Heidelberg, Germany). The reaction was initiated by UV radiation (15 W) and was run for 15 min. The absorbance was measured at a wavelength of 560 nm. The amount of the enzyme that caused inhibition of the NTB reduction reaction by 50% was assumed as a unit of SOD activity (U × mg^−1^ protein). To determine SOD isoenzymes a samples containing 50 μg of protein were separated in a 10% nondenaturing acrylamide gel and visualized using the method of Beauchamp and Fridovich [59].

### 4.16. Catalase Activity

Catalase was assayed using the method of Chance and Maehly [60]. Fresh hyphae (0.250 g) were homogenized in 0.1 M sodium-phosphate buffer (pH 7.0) (Chempur; Piekary Śląskie, Poland). After centrifugation (15,000 *g* for 30 min at 4 °C) the supernatant was collected and used for assays. The assay mixture contained 0.01 M sodium-phosphate buffer (pH 7.0), enzymatic extract, and 3% H_2_O_2_. Absorbance was measured at a wavelength of 240 nm. The catalase activity was expressed as ΔA × min^−1^ × mg^−1^ protein. Catalase isoenzymes were determined by native PAGE on 7.5% acrylamide gels and were localized by the method described earlier by Woodbury et al. [61]. To determine CAT isoenzymes a samples containing 50 μg of protein were separated in a 7.5% nondenaturing acrylamide gel and visualized using the method of Woodbury et al. [61].

### 4.17. Gene Expression Measurement

Hyphae of *P. infestans* (or potato leaves) were frozen in liquid nitrogen and stored at -80 °C until use. The RNA was isolated from 0.150 g of frozen sample using TriReagent (Sigma; Saint-Louis, MO, USA). The obtained RNA was purified with the use of the Deoxyribonuclease Kit (Sigma). For the reverse transcription 1 µg of RNA was processed with the Reverse Transcription Kit (Thermo Scientific Fermentas; Waltham, MA, USA) according to the manufacturer’s instructions. The real-time PCR reactions were performed on a Rotor-Gene 6000 thermocycler (Corbett Life Science; Qiagen; Hilden, Germany). The reaction mixture contained 0.1 µM of each primer (listed in Table S1 in Supplementary Materials), 1 µL of 5× diluted cDNA, 5 µL of Power SYBR Green PCR Master mix (Applied Biosystems; Foster City, CA, USA), and DEPC treated water to a total volume of 10 µL. The PCR reaction initiated denaturation at 95 °C for 5 min. Subsequent stages included 50 cycles consisting of 10 s at 95 °C, 20 s at 53 °C, and 30 s at 72 °C. The reaction was finalized by denaturation at a temperature rising from 72 to 95 °C by one degree every 5 s. The reaction specificity and CT values for individual samples were determined using the real-time PCR Miner Program [62]. The *P. infestans S3a* gene was selected as a reference in *P. infestans* gene expression measurement; the potato *Ef1-α* gene was selected as a reference in expression analysis of the *PiTef1 in planta*. All primers used are presented in Table S1. The relative gene expression was calculated using the Pfaffl mathematical model [63].

### 4.18. Statistical Analysis

All results are based on three biological replicates derived from three independent experiments. For each experiment, means of the obtained values (*n* = 9 or in case of the assessment of the area under disease progress *n* = 15) were calculated along with standard deviations. To estimate the statistical significance between means, the data were analyzed with the use of one-way analysis of variance (ANOVA) followed by a Dunnett’s test at the level of significance α = 0.05 or α = 0.01.

## Supplementary Materials

The following are available online at https://www.mdpi.com/1422-0067/21/21/8375/s1, Table S1. Sequences of primers used for the real-time PCR reaction; Figure S1. The effect of various Cd concentrations on *P. infestans in vitro* growth; Figure S2. The effect of Cd on the selected elements of the enzymatic antioxidant system in *P. infestans*; Figure S3. The original source photo (full-length gel) for Figure 6G showing isoenzyme patterns of SOD; Figure S4. The original source photo (full-length gel) for Figure 6H showing isoenzyme patterns of CAT.

## Abbreviations

CAT	catalase
Cd	cadmium
FW	fresh weight
NO	nitric oxide
8-NO_2_-G	8-nitroguanine
ONOO¯	peroxynitrite
PCO	carbonylated proteins
RNS	reactive nitrogen species
ROS	reactive oxygen species
SOD	superoxide dismutase
TAC	total antioxidative capacity
