# Supplementary material for: Cadmium Stress Reprograms ROS/RNS Homeostasis in Phytophthora infestans (Mont.) de Bary
Source: Int J Mol Sci. 2020 Nov 8;21(21):8375. doi: 10.3390/ijms21218375 (PMC7664633; doi:10.3390/ijms21218375)
Supplement: Supplementary file 1 [file ijms-21-08375-s001.pdf]

Table S1. Sequences of primers used for the real-time PCR reaction.

Number of the gene in NCBI base	Sequence of the primer	GEN	Encoded protein
XM_002901230.1	R: CTCTGGTGGCCGTCTGTAAG<br>F: GGACGCCTTTCCTTCCTTCA	S3a	40S ribosomal protein S3a
XM_002905211.1	R: CCTACGACTACAACGCCCTC<br>F: TTCACGTAAGCCTGGTGGTG	SOD	manganese superoxide dismutase
XM_002898650.1	R: AGTGAATCGCCCACTGAAGG<br>F: TGTTCGGGAAGTAGTTGGGC	CAT	catalase
XM_002999158.1	R: CACGTATTCTCTTCGGCCTG<br>F: TCAACTCCTCATCTGCAAACC	CatG	catalase-peroxidase
XM_002898492.1	R: ACGGATTCATCACCAACGACA<br>F: GCTTGGATGGCTCATGGTTC	GPX	glutathione peroxidase
XM_002898388.1	R: CTTTACCTTCGTGTGCCCCA<br>F: AGGTGCGAGAACTTGGAGTC	PRX	peroxiredoxin-2
XM_0s02898398.1	R: GAGGTGCTGCGTCTGATTGA<br>F: CCTTCTTCCAGTTAGCGGGG	TPX	thioredoxin peroxidase
AB061263	R: ATTGGAAACGGATATGCTCCA<br>F: TCCTTACCTGAACGCCTGTCA	EF1-α	*S. tuberosum* elongation factor 1 alpha
AJ249839	R: ATGACTCGCCTCGGTGATTA<br>F: TCCACACACAAAGTGCATCA	PiTef1	*P. infestans* elongation factor 1 alpha

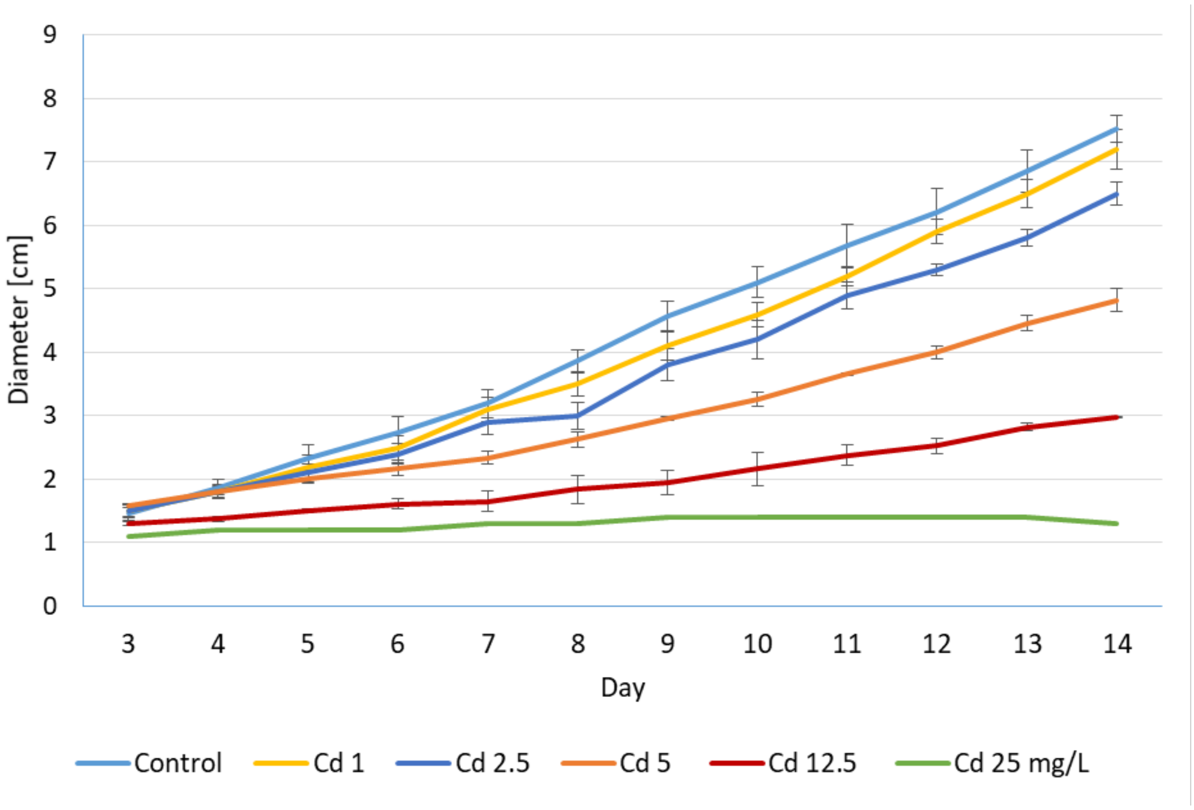

Figure S1. The effect of various Cd concentrations on *P. infestans in vitro* growth. Radial growth of *P. infestans* was measured on medium supplemented with 0 mg/L, 1 mg/L, 2.5 mg/L, 5 mg/L, 12.5 mg/L and 25 mg/L of Cd; The results are an average from three independent experiments ± SD.

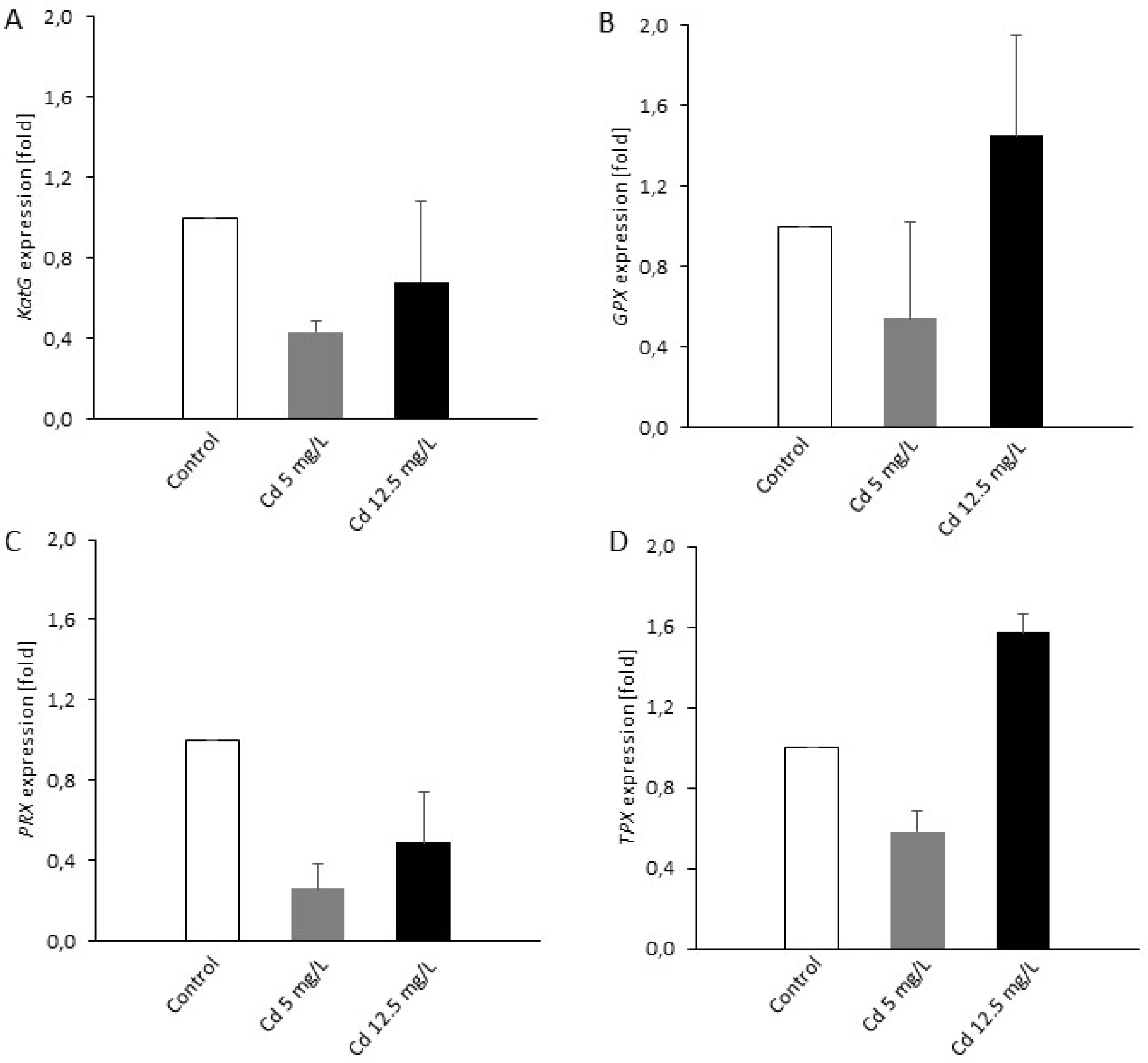

Figure S2. The effect of Cd on the selected elements of the enzymatic antioxidant system in *P. infestans*. RT-qPCR analysis of (A) *CatG*, (B) *GPX*, (C) *PRX* and (D) *TPX* genes expression. The results are an average from three intependent experiments ± SD;

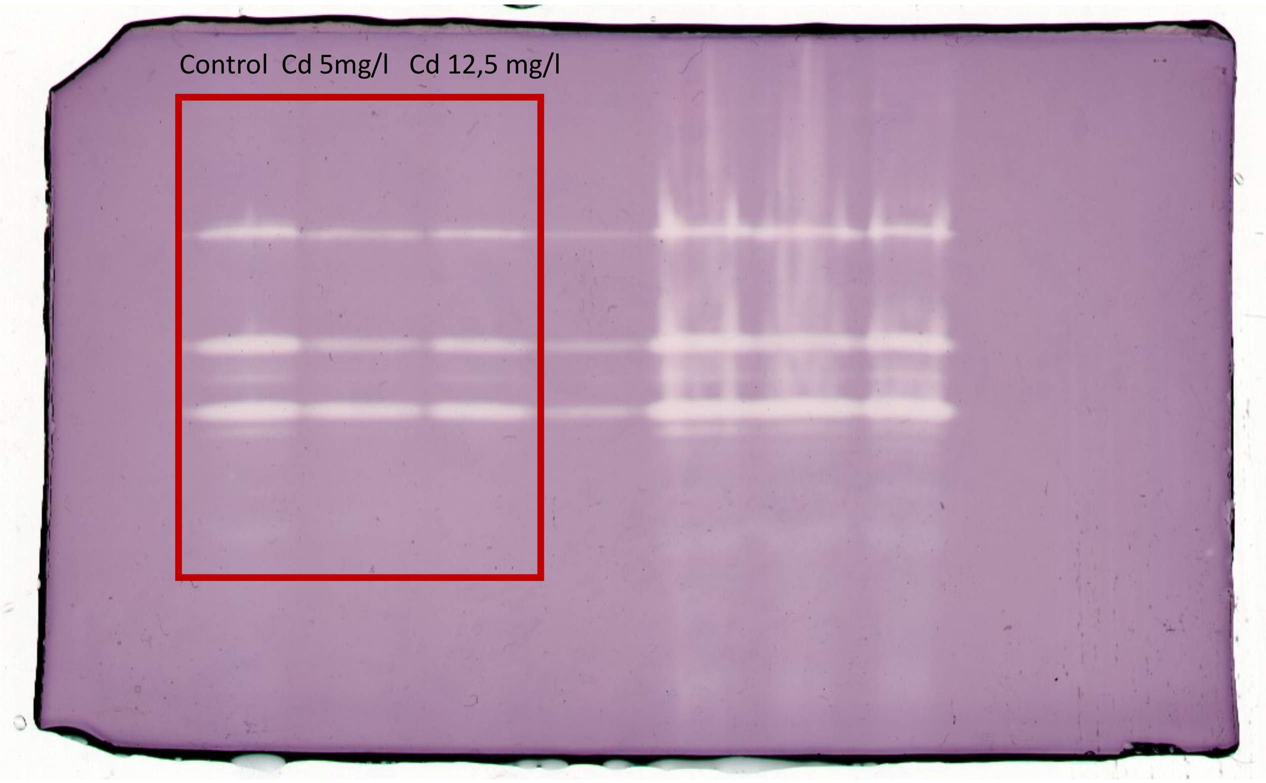

Figure S3. The original source photo (full-length gel) for Figure 6 G showing isoenzyme patterns of SOD. The paths presented in Figure 6 G are marked with a red frame.

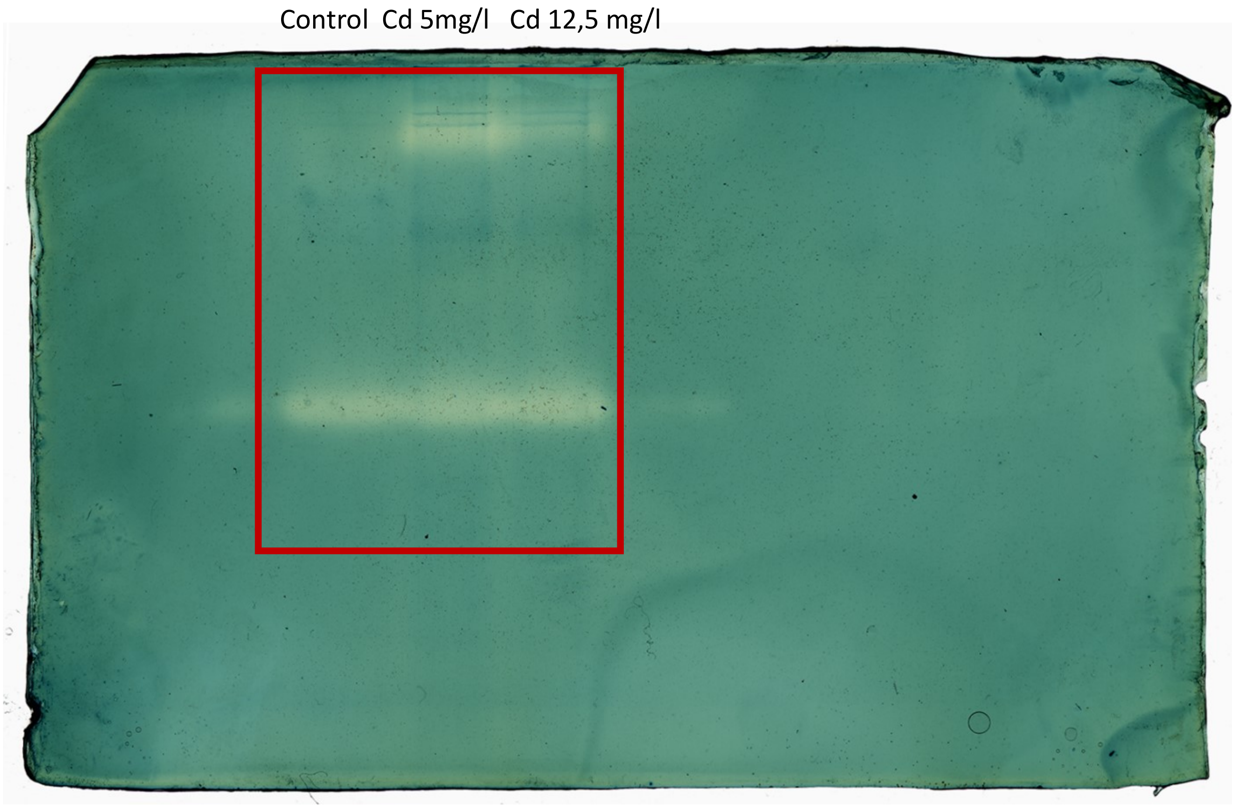

Figure S4. The original source photo (full-length gel) for Figure 6 H showing isoenzyme patterns of CAT. The paths presented in Figure 6 H are marked with a red frame.
